# Characterization of Changes in Polyphenols, Antioxidant Capacity and Physico-Chemical Parameters during Lowbush Blueberry Fruit Ripening

**DOI:** 10.3390/antiox2040216

**Published:** 2013-10-15

**Authors:** Lara Gibson, H. P. Vasantha Rupasinghe, Charles F. Forney, Leonard Eaton

**Affiliations:** 1 Department of Environmental Sciences, Faculty of Agriculture, Dalhousie University, PO Box 550, Truro, NS B2N 5E3, Canada; E-Mails: lara.gibson@dal.ca (L.G.); eatonres@ns.sympatico.ca (L.E.); 2 Atlantic Food and Horticulture Research Center, Agriculture and Agri-Food Canada (AAFC), Kentville, NS B4N IJ5, Canada; E-Mail: charles.forney@agr.gc.ca

**Keywords:** wild blueberry, *Vaccinium angustifolium*, fruit maturity, polyphenols, physico-chemical parameters, antioxidants, organic acids, sugars

## Abstract

Changes in major polyphenols, antioxidant capacity, and selected physico-chemical parameters were examined in lowbush blueberry during fruit ripening. Polyphenols (phenolic acids, flavonols, flavan-3-ols, and anthocyanins), density, soluble solid content, pH, titratable acidity, sugars, organic acids, and antioxidant capacity were determined in fruits of four maturities: green, pink/red, blue, and over-mature. Highest concentrations of flavonols, flavan-3-ols, and phenolic acids were in green fruits: 168 ± 107, 119 ± 29 and 543 ± 91 mg/100 g dry weight (DW) respectively. Highest anthocyanin levels were found in blue and over-mature fruits (1011–1060 mg/100 DW). Chlorogenic acid was the most abundant phenolic acid and quercetin-3-*O*-galactoside the most abundant flavonol in all maturities. Epicatechin was the most abundant flavan-3-ol in green fruits (80 ± 20 mg/100 DW), and catechin was the most abundant in other maturity stages. Increase of glucose and fructose and decrease of organic acids were observed during fruit ripening. Among six organic acids found, quinic acid (1.7–9.5 mg/100 mg DW) was the most abundant throughout the fruit ontogeny. Soluble solids, pH, and density increased with maturity while, titratable acidity decreased. These findings can be helpful in optimizing harvest and processing operations in lowbush blueberry fruits.

## 1. Introduction

Fruits and fruit juices contain larger amounts of polyphenols and are considered as significant sources of health promoting bioactives in human diet [1]. Blueberries in particular are a rich source of polyphenols and as reviewed [2], a serving of ripened blueberries (half a cup) can provide 200–400 mg of total polyphenols (expressed as gallic acid equivalents). Blueberry polyphenols can include flavonoids, condensed and hydrolyzable tannins, stilbenoids, and phenolic acids [3]. Previous studies have shown numerous health benefits like anti-inflammatory, anti-diabetic, and antioxidant effects of blueberry polyphenols. An anthocyanin-rich lowbush blueberry (*Vaccinium angustifolium*) fraction showed hypoglycemic activity in an acute mouse model for type 2 diabetes [4]. In another study, whole blueberry intake reduced the phenotypes of metabolic syndrome in obesity-prone Zucker rats [5]. In the same study, when the diet of Zucker rats were supplemented with blueberries, it was observed that plasma triglycerides, fasting insulin and abdominal fat mass were reduced while adipose and skeletal muscle peroxisome proliferator-activated receptor activity was increased. Consumption of wild blueberry-enriched diet for eight weeks improved the lymphocyte protection against H_2_O_2_-induced DNA damage in Sprague Dawley rats [6]. In a human intervention study, consumption of wild blueberry drink for six weeks significantly reduced the levels of oxidized DNA bases and increased the resistance to oxidatively induced DNA damage [7]. All in all it suggests health promoting and disease preventing ability of blueberry polyphenols.

Lowbush blueberries are one of Nova Scotia, Canada’s important commercial agricultural crops and produces about 18 million kilograms of berries annually, which are sold to both domestic and export markets [8]. Processing of blueberries often includes hydro-density sorting to divert green, red, and over-mature maturity stages from being included in the packaging of fresh or frozen blueberries. A significant proportion from mechanically harvested fruits consists of unripe fruits and these would be a waste product in the blueberry processing industry. As each phenolic class contributes to different traits in the berry such as astringency or colour, the composition of polyphenols is expected to change with maturity. The polyphenolic content of blueberries has been characterized among and within species and some subclasses have been examined in relation to maturity. Influence of maturity on blackberry polyphenols and physico-chemical parameters has been previously reported [9]. Phenolic content of highbush blueberries during ripening was also previously documented but reports on changes in polyphenol classes and physico-chemical changes during lowbush blueberry ripening is scarce to our understanding. Characterizing the physico-chemical nature of berries as they mature could provide useful information to optimize harvest maturity and better utilization of mechanically harvested fruits which consist of significant proportion of unripe fruits. Investigation of the polyphenol composition of unripe or other unmarketable maturity stages of lowbush blueberry fruits provide insights for alternative use of rejected fruits. With this background information, the current study was carried out, with the objective of understanding physico-chemical and polyphenols composition changes that take place over lowbush blueberry fruit maturity and testing polyphenols as ripening markers of the fruits.

## 2. Materials and Methods

### 2.1. Plant Materials and Chemicals

Three distinct clones were marked at one location, Debert, Nova Scotia, Canada, and fruits were harvested from July to October, 2007. The clones consisted of one unnamed wild clone “Chignecto”, and two planted clones: a hybrid of “79-12” and “Brunswick”. These clones were chosen for their large size to ensure sufficient quantities of berries available for all maturity classes. All three clones were wild pollinated and managed with regular industry practices by the Wild Blueberry Institute of Nova Scotia.

Approximately 500 g of fruit from each of four maturity classes, green, pink/red, blue and over-mature (senescent fruit that expressed splitting, softening or shriveling), were hand harvested during the season. As the maturity classes follow in succession, only a portion of the berries (15 g to 100 g of each maturity class) were collected at one time to ensure subsequent maturity classes would develop on the clone. Collection dates were at least weekly starting in July and increasing to daily in September and October. Following each harvest, fruits were transported in coolers to the Dalhousie University Agricultural Campus (Truro, NS, Canada). Five replicates (20 g each) of fresh fruits from each maturity stage were processed for density using water displacement. The remaining samples were immediately frozen at −20 °C. Chemical analysis was conducted on the composite frozen fruit samples collected over time to determine polyphenol profiles, sugar, and organic acid content, antioxidant capacity, titratable acidity, soluble solid content, and pH.

The liquid chromatography standards used for the polyphenol analysis included: caffeic acid (Caf), ferulic acid (Fer), quercetin-3-*O*-rutinoside (Q3-Rut), epigallocatechin (EGC), catechin (Cat), epicatechin (Epicat), cyanidin-3*-O*-glucoside (C3-Glu), petunidin-3-*O*-glucoside (P3-Glu), petunidin-3-galactoside (P3-Gal), delphinidin-3-*O*-glucoside (D3-Glu), delphinidin-3*-O*-galactoside (D3-Gal), malvidin-3*-O*-glucose (M3-Glu), and malvidin-3-*O*-galactoside (M3-Gal), purchased from ChromaDex (Santa Ana, CA, USA). Quercetin-3*-O*-galactoside (Q3-Gal) and quercetin-3-*O*-rhamnoside (Q3-Rha) were purchased from Indofine Chemical Co. (Hillsborough, NJ, USA). Quercetin-3*-O*-glucoside (Q3-Glu), chlorogenic acid (Chl), gallic acid, sodium carbonate, sodium acetate trihydrate, 2,4,6-tris(2-pyridyl)-S-triazine (TPTZ), Trolox, fluorescin, Folin-Ciocalteu reagent, fluorescein sodium salt, ferric chloride, and phosphate buffer were purchased from Sigma-Aldrich (St. Louis, MO, USA) and cyanidin-3-*O*-galactoside (C3-Gal) from Extrasynthese Inc. (Paris, France). 2,2′-Azobis (2-amidinopropane) dihydrochloride (AAPH) was purchased from Walco Chemical Products Co. Inc. (Buffalo, NY, USA). Hydrochloric acid and 96-well microplates were purchased from Fisher Scientific Inc. (Ottawa, ON, Canada).

### 2.2. Sample Preparation for Analysis

Three replicate samples of 50 g each were taken from the composite frozen fruit sample collected over time for each clone and were used to determine the polyphenol profiles, antioxidant capacity, sugars, and organic acids. The berry samples were freeze dried for 60 h in a freeze dryer (model 2085C0000, Kinetics Thermal Systems, Stone Ridge, NY, USA). The temperature was kept at −25 °C for the first 24 h and then the berries were removed, partly crushed, and returned to the freeze dryer. Afterwards, the temperature was held at −25 °C for five hours and then raised to 5 °C for 16 h, and finally, raised to room temperature for the remaining time.

Following drying, fruits were weighed to determine the percent moisture and then ground using a coffee grinder (model DCG 12BCC, Cuisinart, Woodbridge, ON, Canada). Approximately 0.3 g of powdered fruit was mixed with 15 mL of methanol: acetone: water: formic acid extraction solution (40:40:20:0.1, v/v). Samples were sonicated (model 750D, VWR Intl. Ltd., Montreal, QC, Canada) for 15 min × 3 times with 10 min intervals between sonications and then centrifuged (model Durafuge 300, Precision Scientific, Richmond, VA, USA) at 5000 rpm for 15 min. Two millilitres of the supernatant were filtered through a 0.22 µm nylon filter before analysis by high performance liquid chromatography (HPLC).

### 2.3. HPLC Mass Spectrometry Analysis

Concentrations of phenolic acids, flavan-3-ols, flavonols, and anthocyanins were determined using HPLC coupled to electrospray ionization and triple quadrupole mass spectrometry as described by Rupasinghe *et al*. [10]. Electrospray ionization in negative ion mode was used for flavonols, flavan-3-ols and phenolic acids in the multiple reaction mode of mass spectrometric analysis as follows: Q3-Rut *m*/*z* 448→301, Q3-Gal and Q3-Glu *m*/*z* 463→301, Q3-Rha *m*/*z* 447→301, Chl *m*/*z* 353→191, Caf *m*/*z* 179→35, Fer *m*/*z* 193→134, EGC *m*/*z* 457→169, Cat *m/z* 289→109, and Epicat *m*/*z* 290→109. Positive ion mode electrospray ionization was used for the anthocyanins as follows: C3-Glu *m*/*z* 448.8→286.8, C3-Gal *m*/*z* 449→287, D3-Glu *m*/*z* 465→302.6, D3-Gal *m*/*z* 465→302.6, P3-Glu and P2-Gal *m/z* 479→316.8, M3-Glu and M3-Gal *m*/*z* 493→330.8.

### 2.4. Antioxidant Capacity Assays

The prepared sample extracts were used in two assays: Ferric Reducing Antioxidant Power (FRAP) and Oxygen Radial Absorbance Capacity (ORAC) assays to measure total antioxidant capacity, exactly as described by Huber and Rupasinghe [11].

### 2.5. Determination of Sugar and Organic Acid Profiles

Freeze dried berry powder (1.5 g) was mixed with 15 mL of above mentioned extraction solvent and sonicated for 15 min twice with a 10 min interval between sonications. An 8 mL aliquot was taken and the solvent was complete dried (UV vacuum system, Thermo Electra Corporation, Waltham, MA, USA). The sample was re-dissolved in 5 mL of water and vacuum filtered through a C-18 Bond Elut 500 mg SEP cartridge (Varian Canada Inc., Mississauga, ON, Canada). Approximately 1.5 mL of elute was filtered through a 0.22 µm polytetrafluoroethylene (PTFE) nylon syringe filter and split for sugar and acid analysis. The filtered extract was analyzed for sugar using as described by Forney *et al*. [12]. The organic acid content was determined using a Beckman System Gold HPLC with a Synergi Hydro-RP (250 × 4.6 mm, 4 µm) column (Phenomenex Inc., Torrance, CA, USA) and a photodiode-array detector using 0.01 M H_2_SO_4_ (pH 2.5) as the mobile phase at a flow rate of 0.5 mL/min.

### 2.6. Determination of Physico-Chemical Properties

Soluble solid content, titratable acidity, and pH were determined from juice extracted from the composite samples. Three replicates for each clone and each maturity stage were taken totaling 36 samples. Each replicate (100 g) was thawed for four hours and squeezed through a cheesecloth lined garlic press. Soluble solid content was measured with a digital refractometer (Model 300016, Sper Scientific, Scottsdale, AZ, USA), titratable acidity with an automated titrator (785 DMP Titrino, Metrohm, Riverview, FL, USA) and pH with a pH meter (Accumet model 10, Denver Instruments Company, Bohemia, NY, USA).

### 2.7. Statistical Analysis

To allow comparisons across groups and among clones, polyphenol results were expressed on a dry weight (DW) basis (mg/100 g) and sugar and acid data were converted to mg/100 mg DW. The data was normalized using log transformations. The statistics program SYSTAT 10 (SYSTAT, Chicago, IL, USA) was used for multivariate analysis of variance within the polyphenol classes and for analysis of variance (ANOVA) for individual compounds and sub-class totals. Changes in the polyphenol class totals across maturity were examined by fixing the clone variable and pooling the compounds in each of the four phenolic compound classes, flavan-3-ols, flavonols, phenolic acids, and anthocyanins. SYSTAT 13 (SYSTAT, Chicago, IL, USA) was used for ANOVA for sugar, acid, total phenolics, total anthocyanins and physico-chemical measures. Changes across maturity within each compound were examined using ANOVA and differences among maturity stages were determined using Tukeys *post hoc* test.

## 3. Results

### 3.1. HPLC/MS Analysis of Phenolic Composition

In this study, change in the polyphenols composition was examined as markers during fruit development and ripening. As fruits ripened (from green to blue), total quantified phenolic acids and flavan-3-ols significantly decreased while total anthocyanins significantly increased (Table 1). Therefore, these sub classes of polyphenols have a potential to be used as markers during fruit maturity. Total quantified flavonols significantly changed when fruits matured from red to blue. Overall, the total phenolic content remained unchanged during fruit maturity and ripening.

**Table 1:** The major polyphenols (mg/100g DW) found in lowbush blueberries at four maturities.

		Maturity				Comparison among clones
		Green	Red	Blue	Over mature	
**Flavonol**	Q3-Rutinoside	30 ± 41 **^a A^**	10 ± 13 **^a A^**	6 ± 7 **^a A^**	5.4 ± 4.1 **^a A^**	(1,2) 3
	Q3-Galactoside	107 ± 50 **^a B^**	81 ± 32 **^a B^**	50 ± 17 **^b B^**	37 ± 10 **^b B^**	1 (2,3)
	Q3-Glucoside	25 ± 17 **^a A^**	15 ± 7 **^a C^**	10 ± 4 **^b C^**	7 ± 2 **^b A^**	(1,2) 3
	Q3-Rhamnoside	6 ± 5 **^a A^**	2 ± 2 **^a A^**	1 ± 1 **^a A^**	3 ± 3 **^a A^**	(1,3) 2
	**Total quantified Flavonols**	168 ± 107 **^a^**	108 ± 45 **^a^**	66 ± 22 **^b^**	52 ± 7 **^b^**	1 (2,3)
**Phenolic Acids**	Chlorogenic	533 ± 91 **^a A^**	307 ± 32 **^b A^**	200 ± 25 **^c A^**	182 ± 6 **^c A^**	(1,2,3)
	Caffeic	4 ± 3 **^a B^**	2 ± 1 **^b B^**	1 ± 0.3 **^b B^**	0.8 ± 0.1 **^b B^**	1 (2,3)
	Ferulic	7 ± 4 **^a B^**	2 ± 1 **^b B^**	1 ± 0.3 **^b B^**	1 ± 0.2 **^b C^**	1 (2,3)
	**Totals quantified Phenolic acids**	543 ± 91 **^a^**	311 ± 33 **^b^**	202 ± 25 **^c^**	184 ± 6 **^c^**	(1,2,3)
**Flavan-3-ols**	ECG	0.8 ± 0.1 **^a A^**	0.5 ± 0.1 **^b A^**	0.4 ± 0.3 **^a A^**	1 ± 0.5 **^c A^**	(1,2,3)
	Catechin	39 ± 13 **^a B^**	9 ± 3 **^b B^**	5 ± 2 **^b B^**	6 ± 4 **^b B^**	1 (2,3)
	Epicatechin	80 ± 20 **^a C^**	7 ± 2 **^b B^**	2 ± 1 **^c C^**	5 ± 2 **^d B^**	(1,2,3)
	**Total quantified Flavan-3-ol**	119 ± 29 **^a^**	16 ± 5 **^b^**	8 ± 3 **^c^**	12 ± 6 **^c^**	(1) (2) (3)
**Anthocyanins**	Cy-3-Glucoside	2 ± 0.3 **^a A^**	59 ± 20 **^b A^**	76 ± 52 **^b A^**	55 ± 45 **^b A^**	(1,2,3)
	Cy-3-Galactoside	2 ± 1.3 **^a A^**	68 ± 9 **^b A^**	69 ± 29 **^b A^**	53 ± 30 **^b A^**	(1,2,3)
	Del-3-Glucoside	0.9 ± 0.1 **^a B^**	81 ± 23 **^b A^**	151 ± 90 **^b B^**	122 ± 87 **^b B^**	(1,2,3)
	Del-3-Galactoside	0.9 ± 0.1 **^a B^**	96 ± 12 **^b A^**	163 ± 56 **^c B^**	135 ± 62 **^c B^**	(1,2,3)
	Pet-3-Glucoside	0.4 ± 0.1 **^a C^**	116 ± 24 **^b A^**	261 ± 151 **^c B^**	244 ± 159 **^b B^**	(1,2,3)
	Pet-3-Galactoside	0.3 ± 0.1 **^a C^**	87 ± 22 **^b A^**	179 ± 57 **^c B^**	188 ± 72 **^d B^**	(1,2,3)
	Mal-3-Glucoside	0.7 ± 0.1 **^a D^**	34 ± 11 **^b B^**	95 ± 48 **^c A^**	110 ± 58 **^c B^**	(1,2,3)
	Mal-3-Galactoside	0.7 ± 0.1 **^a D^**	24 ± 10 **^b B^**	66 ± 19 **^c A^**	103 ± 30 **^d B^**	(1,2,3)
	**Total quantified Anthocyanin**	8 ± 2 **^a^**	565 ± 70 **^b^**	1060 ± 490 **^c^**	1011 ± 534 **^c^**	(1) (2) (3)
**Total**	**Total phenolics by HPLC/MS**	839 ± 127 **^a^**	999 ± 77 **^a^**	1335 ± 501 **^a^**	1259 ± 542 **^a^**	1 (2,3)

Results are expressed as mean ± standard deviation. Superscript letters indicate Tukeys mean comparison results within rows (lower case) and within columns (upper case); Different letters indicate significant difference at *p* < 0.05; Numbers contained within brackets indicate clones that were not significantly different at *p* < 0.05. *n* = 9 (mean of triplicates of three clones); Table abbreviations: Q (quercetin), EGC (epigallocatechin), Cy (cyanidin), Del (delphinidin), Pet (petunidin), and Mal (malvidin).

When considering the composition of polyphenols in different maturity stages, phenolic acids made up 65%, flavonols 20%, flavan-3-ols 14%, and anthocyanins 1% of the measured polyphenols in green fruits. The majority of polyphenols in red fruits were anthocyanins (56%), followed by phenolic acids (31%), flavonols (11%) and flavan-3-ols (2%). Blue and over mature fruits were similar with concentrations of 79%–80% anthocyanins, 15% phenolic acids, 4%–5% flavonols, and 1% flavan-3-ols. Differences within all compounds were affected by both clone and maturity (*p* < 0.0001) but no significant interaction was evident among them (*p* > 0.05) (data not shown).

Within each polyphenol class, individual compounds varied substantially during fruit ripening (Table 1). Chlorogenic acid was the most abundant phenolic acid in all maturities, being approximately 100 times higher in concentration than either caffeic or ferulic acids. Among the flavonols, quercetin-3-*O*-galactoside was the most abundant in all maturity stages, with concentrations being more than twice as high as other flavonols. Epicatechin was the most abundant flavan-3-ol in green fruit, whereas catechin was the most abundant in other maturity stages. However, both compounds decreased significantly as fruit matured. In green fruit, cyanidin galactoside and glucoside were the most abundant anthocyanins whereas glucosides and galactosides of petunidin and delphinidin were most abundant in other maturities.

### 3.2. Antioxidant Capacity

The results of Ferric Reducing Antioxidant Power (FRAP) assay showed a significantly higher antioxidant capacity (*p* < 0.05) for green berries (Table 2). Contrastingly, results of Oxygen Radical Absorbance Capacity (ORAC) assay showed that green berries showed lower ORAC values compared to the other maturities indicating a lower antioxidant capacity. The other three maturity stages showed no significant difference when measured by both ORAC and FRAP.

**Table 2:** Total phenolic content and antioxidant capacity of lowbush blueberries at four maturity stages.

Antioxidant capacity assay	Maturity				Comparison among clones
	Green	Red	Blue	Over mature	
FRAP (mg TE/g DW)	302 ± 97 ^a^	151 ± 23 ^b^	125 ± 62 ^b^	160 ± 60 ^b^	(1,3) 2
ORAC (µmol TE/g DW)	57 ± 0.4 ^a^	195 ± 28 ^b^	165 ± 51 ^b^	206 ± 45 ^b^	(1,2,3)

Results are expressed as mean ± standard deviation; Superscript letters within rows indicate significant difference at *p* < 0.05 based on the Tukeys *post hoc* test; Numbers contained within brackets indicate clones that were not significantly different at *p* < 0.05; Abbreviations: FRAP (Ferric Reducing Antioxidant Power), ORAC (Oxygen Radical Absorbance Capacity), TE (Trolox Equivallents).

Each antioxidant capacity assay suggested different relationships between the clones and antioxidant capacity (data not shown). Results from ORAC assay suggested no significant difference between clones and antioxidant capacity (*p* > 0.05) whereas according to results of FRAP assay, antioxidant capacity of clone two was significantly different from the rest (*p* < 0.05).

**Table 3:** Sugar, organic acid content and selected physico-chemical measures of four maturity stages in lowbush blueberries.

		Maturity Stage				Comparison among clones
		Green	Red	Blue	Over mature	
**Sugars**	Glucose	4.2 ± 6.8 ****^a^****	13.2 ± 2.0 **^b^**	17.8 ± 3.4 **^b^**	16.9 ± 1 **^b^**	(1,2,3)
**(mg/100 mg DW)**	Fructose	4.2 ± 6.7 **^a^**	13.3 ± 1.7 **^b^**	18.1 ± 3.3 **^b^**	19.4 ± 1.5 **^b^**	(1,2,3)
	Galactose	0.02 ± 0.02 **^a^**	0.02 ± 0.01 **^a^**	0.05 ± 0.02 **^a^**	0.06 ± 0.04 **^a^**	(1,2,3)
	Sucrose	0.01 ± 0.03 **^a^**	0.1 ± 0.03 **^a^**	0.07 ± 0.02 **^a^**	0.14 ± 0.03 **^b^**	(1,2,3)
	**Total Sugars (%DW)**	**8.5 ± 13.4 ^a^**	**26.6 ± 3.6 ^b^**	**36.0 ± 6.77 ^b^**	**36.5 ± 2.5 ^b^**	(1,2,3)
**Acids**	Quinic acid	9.5 ± 2.9 **^a^**	4.9 ± 0.3 **^a^**	2.9 ± 0.5 **^b^**	1.7 ± 0.2 **^c^**	(1,2,3)
**(mg/100 mg DW)**	Citric acid	0.8 ± 0.7 **^a^**	1.8 ± 0.7 **^b^**	1.2 ± 0.1 **^b^**	0.6 ± 0.1 **^a^**	(1,2) 3
	Malic acid	0.5 ± 0.1 **^a^**	0.3 ± 0.1 **^a^**	0.1 ± 0.01 **^b^**	0.4 ± 0.1 **^a^**	(1,2,3)
	Tartaric acid	ND	Trace	Trace	Trace	NA
	Shikimic acid	0.1 ± 0.06 **^a^**	0.04 ± 0.01 **^b^**	0.02 ± 0.01 **^b^**	0.01 ± 0.003 **^b^**	(1,3) 2
	Succinic acid	0.2 ± 0.1 **^a^**	0.2 ± 0.06 **^a^**	0.1 ± 0.05 **^a^**	0.1 ± 0.06 **^a^**	(1,3) 2
	**Total Acids (%DW)**	**11.1 ± 3.1 ^a^**	**7.2 ± 0.7 ^b^**	**4.4 ± 0.5 ^c^**	**2.8 ± 0.32 ^d^**	
**Physico-chemical measures**	pH	3.2 ± 0.03 **^a^**	3.2 ± 0.1 **^a^**	3.3 ± 0.1 **^b^**	3.63 ± 0.1 **^c^**	(1,2,3)
	TA (citric acid equivalents, g/L)	0.2 ± 0.02 ^a^	0.1 ± 0.02 ^b^	0.06 ± 0.01 ^c^	0.04 ± 0.01 ^d^	(1,2,3)
	SSC (°Brix)	7 ± 0.4 **^c^**	8 ± 0.3 **^b^**	10 ± 0.8 **^a^**	11 ± 2 **^a^**	(1,2,3)
	Density (mg/mL)	0.98 ± 0.03 **^a^**	1.00 ± 0.03 **^b^**	1.03 ± 0.02 **^c^**	1.02 ± 0.02 **^c^**	(1,2,3)

Values are expressed as means ± standard deviations; Superscript letters within rows indicate significant difference at *p* < 0.05 based on the Tukeys *post hoc* test. Numbers contained within brackets indicate clones that were not significantly different at *p* < 0.05. Abbreviations: SSC = soluble solid content, TA = Titratable acidity, ND = not detected, NA = not applicable.

### 3.3. Sugars and Organic Acid Profiles

In all maturity stages, glucose and fructose were the most abundant sugars where galactose and sucrose were found only in trace amounts (Table 3). The amount of these two sugars was significantly lower in green fruits compared to other maturities (4.2 ± 6.8 and 4.2 ± 6.7 mg/100 mg dry weight respectively). There was no significant difference in glucose and fructose between the other three maturity stages (*p* > 0.05). Total sugar content increased during fruit maturity where green fruits contained total sugars as low as 8.5% and increased to 36% in blue and over-mature fruits. No significant differences were observed among clones for the analyzed sugars (*p* > 0.05).

Five organic acids were detected in lowbush blueberries, (−)—quinic, citric, L (−)—malic, (−)—shikimic, and succinic (Table 3). L (+)—tartaric acid was not detected in green fruit, but traces were found in red, blue and over-mature fruits (Table 3). Quinic acid was the most abundant in fruits of all maturities, followed by citric acid and the concentration of malic, shikimic, and succinic acids were less than 1%. Total percent acids decreased as berries ripened, falling from 11.1 ± 3.1 mg/100 mg DW in green fruit to 7.2 ± 0.7 mg/100 mg DW in red fruit, 4.4 ± 0.5 mg/100 mg DW in blue fruit, and 1.7 ± 0.2 mg/100 mg DW in over-mature fruit. There was no significant difference among clones for quinic, malic, or total acids (*p* > 0.05). For citric acid, clone 3 was different from clones 1 and 2 (*p* < 0.05) and for both shikimic and succinic acid, clone 2 was different from clones 1 and 3 (*p* < 0.05).

### 3.4. Physico-Chemical Parameters

Both soluble solid content and fruit density increased as fruits matured (Table 3). Both the parameters did not significantly change when blue fruits were turned to over-mature fruits. The pH changed during fruit ripening where pH of green and red fruits were significantly lower (3.2) than pH of blue (3.3) and over-mature (3.63) fruits. Titratable acidity decreased significantly (*p* < 0.05) from 0.2 g/L citric acid equivalents in green fruits to 0.04 g/L citric acid equivalents in over-mature fruits.

## 4. Discussion

The current study focused on how polyphenol composition and different physico-chemical parameters changed during lowbush blueberry fruit ripening. The total phenolic content was constant during fruit maturation when measured by HPLC/MS, suggesting constrained biosynthetic pathways. Although the total phenolic content was constant during fruit maturation, the composition of polyphenols changed, resulting in a change in astringency and colour. Flavan-3-ols made up 14% of the total phenolics in the green berries, and only 1%–2% in subsequent maturity stages. Anthocyanin concentration increased about 70-fold between green and red berries. This pattern of decreasing non-anthocyanin polyphenols and increasing anthocyanins has been noted in other *Vaccinium* species, such as highbush blueberry, cranberries (*Vaccinium macrocarpon* Ait.), and bilberry (*Vaccinium myrtillus* L.) [13,14,15]. As explained by Kalt and others [16], there is a shift in the pool of total phenolics towards anthocyanin synthesis and an overall decrease in the synthesis of other polyphenols during highbush blueberry ripening. The same phenomenon can be applied to lowbush blueberry as well.

To best of our understanding, the quantification of individual compounds throughout maturation and senescence has not been previously reported for lowbush blueberry fruits. Ripe blueberries were reported to contain 15 anthocyanins, but the most abundant were the glucosides and galactosides of delphinidin, malvidin, petunidin, cyanidin, and peonidin [17]. Although significant differences were observed among total anthocyanin levels at all maturities, no single anthocyanin was more abundant than the others in the current study. Though there was no significant difference among clones for any individual anthocyanin, total anthocyanins were significantly different among three clones. Similarly, Clonal differences in fruit colour have been observed in ripe lowbush blueberries [17]. The most abundant flavonol in all maturity stages was quercetin-3-*O*-galactoside. Similar findings were previously reported on other *Vaccinium* species [18]. Quercetin compounds in *Vaccinium* species could function as anthocyanin co-pigments or free radical scavengers [17,19]. Phenolic acids were the most abundant group of polyphenols and among three phenolic acids, chlorogenic acid was the most abundant at all maturities. Similar findings were reported previously [13]. Further, chlorogenic acid can act as an anthocyanin co-pigment increasing the colour intensity of anthocyanins between pH 2–7 [20] and also act as a free radical scavenger [21]. Oligomeric polyphenols like proanthocyanidins in blueberry were reported in literature [22] but the scope of this study was limited to investigations on monomeric polyphenols. Further investigations are warranted in biochemical mechanism(s) underlying the ripening-dependent changes in polyphenol profiles.

There has been a great deal of interest in the antioxidant content of blueberry fruit as the high antioxidant capacity has been correlated with health benefits [23]. The assays used in the current study measures antioxidant capacity through different properties; ORAC was based on hydrogen atom transfer (HAT), FRAP was based on a single electron transfer (SET) [24]. In the current study, FRAP assay indicated that green fruits had significantly higher antioxidant capacity than the rest. Among several antioxidant capacity measurement assays, FRAP assay showed the highest correlation with total phenolics [25]. The antioxidant assays as well as total phenolics measured by HPLC were not significantly different among red, blue and over-mature fruits. These results suggested that the antioxidant capacity at each maturity resulted from the presence of different compounds, which may react differently to the antioxidant assays. Contrasting findings were reported on different types of berries and antioxidant and total phenolic levels during fruit maturity. In highbush blueberries, total phenolic content and ORAC decreased during fruit maturity [16]. Both blackberry and strawberry ORAC values decreased as fruit matured [26]. In raspberries (*Rubus idaeus* L.), ORAC increased with maturity [26] which was similar to the findings in the current study.

When considering the sugar profile, glucose, fructose, sucrose, and galactose were found in lowbush blueberry fruits at all maturities. Glucose and fructose were the most abundant sugars, with sucrose and galactose contributing less than 0.2 mg/100 mg DW. Glucose and fructose concentrations increased as berries turned from green to red and then changed little as fruit further ripened and senesced. Contrastingly, Kalt and MacDonald [27] reported glucose and fructose concentrations increased in over-ripe fruit, whereas the current findings reported no change between blue and over-ripe fruits.

Organic acids influence fruit flavour [28], and the concentration of organic acids may affect perceived sweetness [29]. Quinic acid was the most abundant in fruit of each maturity, comprising between 65% and 85% of all organic acids. Citric and malic acids were the next most abundant, followed by succinic and shikimic. Quinic acid is a precursor to chlorogenic acid [30]. In the current study, the high levels of quinic acid may relate to the high levels of chlorogenic acid, as both show similar distribution among maturities. The high levels of quinic acid and low levels of citric, malic, tartaric, shikimic and succinic acid were found in all three clones and no significant difference in quinic acid concentrations among clones were observed. Citric acid increased in fruit as they turned from green to red, and decreased as fruit became over-mature. These findings disagree with the findings of Kalt and MacDonald [27], where citric acid decreased as fruit turned blue and then increased in over-ripe fruit. Malic acid decreased as fruit changed from green to blue and then increased in over-ripe fruit, while Kalt and MacDonald [27] found a continuous decrease in malic acid as fruit ripened and senesced. The decrease in acidity and increase in sugar concentrations as fruit ripened would result in a sweeter flavour [29]. In blueberries the decrease in organic acids is thought to result from organic acid catabolism, paired with dilution due to increased fruit size [31]. Titratable acidity decreased while soluble solid content increased during fruit ripening indicating the reduction of acidity and increase in sweetness in the ripe fruit compared to the immature fruits. Similar trend of titratable acidity and soluble solid content results were reported for blackberry during ripening [9].

Lowbush blueberry clones are phenotypically and genotypically variable [32]. The three clones that were examined in this study had significant differences in their fruit constituents such as flavonols, phenolic acids, flavan-3-ols, total phenolics, and organic acids. These results agree with the previous findings by others [27,33]. There were no differences among clones for anthocyanins, sugars, soluble solid content, density, pH, or titratable acidity, which contrasts the results of Kalt and MacDonald [27] who found significant differences in these measures among clones. However, density, pH and titratable acidity are all related to the amount of sugar found in the fruit and therefore it is not unexpected that where significant differences are found among clones in the sugar levels, there would also be significant differences in the associated physico-chemical properties, or as is the case in this study that no significant difference in sugar levels among clones, also results in no significant difference among clones in soluble solid content, density, pH, or titratable acidity.

## 5. Conclusions

Optimization of harvesting time and valorization of processing waste are distinct problems for lowbush blueberry industry. Therefore, this study investigated the change in phenolic composition and several physico-chemical parameters during lowbush blueberry fruit maturation. Concentrations of flavonols, flavan-3-ols, and phenolic acids decreased with maturity, while anthocyanins increased. Glucose and fructose concentrations increased, while organic acid concentrations decreased as fruits matured. The physico-chemical measures of soluble solid content, pH and density increased with maturity, while titratable acidity decreased. Therefore, none of these measures seems to be independently used to measure blueberry fruit maturity. However, further investigations of non-destructive methods to analyze individual polyphenols during fruit maturity may help in determining the optimal time to harvest that could minimize processing waste. The present data also indicate that not marketable green, red, and over-mature fruits that are rejected from the blueberry processing have a potential for use in developing value-added food ingredient or natural health products.
